# The “sweet- and sour-spot” of occupational physical activity for back pain: a prospective accelerometer study among eldercare workers

**DOI:** 10.5271/sjweh.4170

**Published:** 2024-07-01

**Authors:** Stavros Kyriakidis, Charlotte Lund Rasmussen, Karen Søgaard, Andreas Holtermann, Charlotte Diana Nørregaard Rasmussen, Nidhi Gupta

**Affiliations:** 1 Department of musculoskeletal disorders and physical workload, National Research Centre for the Working Environment, Copenhagen, Denmark.; 2 Department of Sports Science and Clinical Biomechanics, University of Southern Denmark, Odense, Denmark.; 3 Curtin School of Allied Health, Faculty of Health Sciences, Curtin University, Australia.

**Keywords:** compositional data analysis, musculoskeletal health, occupational health, physical behavior, time-use epidemiology

## Abstract

**Objectives:**

Both high and low levels of occupational physical activity are associated with back pain. Thus, there might be a “sweet- and sour-spot” of occupational physical activity for back pain. Our aim was to investigate if there exists an occupational physical activity “sweet- (lowest risk) and sour-spot” (highest risk) for back pain.

**Methods:**

A total of 396 eldercare workers from 20 Danish nursing homes participated. Occupational physical activity was measured between 1–4 working days using thigh-worn accelerometry. Back pain intensity was reported monthly on a scale from 0–10 over 1-year. A zero-inflated mixed-effects model was developed regressing occupational physical activity against back pain, adjusted for confounders. The “sweet- and sour-spot” were defined as the occupational physical activity compositions (sitting, standing, light, and moderate-to-vigorous) associated with the 5% lowest and highest risk for back pain, respectively.

**Results:**

The composition associated with the lowest risk of back pain – the “sweet-spot”– consisted of 71% worktime spent sitting, 18% spent standing, 5% spent on light physical activity and 6% spent on moderate-to-vigorous physical activity. The composition associated with highest risk for back pain –the “sour-spot”– consisted of 8% worktime spent sitting, 66% spent standing, 4% spent on light physical activity, and 21% spent on moderate-to-vigorous physical activity.

**Conclusions:**

The “sweet-spot” of occupational physical activity for back pain among eldercare workers involves more sitting and light physical activity time, while the “sour-spot” involves more standing and moderate-to-vigorous physical activity time. Research on the occupational physical activity “sweet- and sour-spot” is needed.

Eldercare work is a profession facing recruitment and retention challenges globally (1). The staffing shortage in eldercare work can be attributed to low pay, poor working conditions and lack of recognition at the societal level (2). Another factor that hinders a long and sustainable employment among eldercare workers is musculoskeletal pain, and especially back pain, with an estimated 1-year prevalence between 51–71% (3). Back pain is known to be associated with reduced work ability (4), sickness absence (5, 6) and early retirement (7). In addition, back pain is a major barrier for performing work tasks, such as resident handlings, with 9% of eldercare workers stating that they feel limited to perform their work due to pain (8).

Work-related risk factors, such as working in awkward postures and manual handlings of residents have been found to be some of the main contributors of back pain among eldercare workers (9, 10). However, recently high occupational physical activity, such as prolonged walking and standing time, has also shown to increase the risk of back pain (11, 12). On the other end of the spectrum, prolonged sitting has also been associated with increased risk of back pain (13, 14). But when occupational physical activities either become “too much” or “too little” for developing back pain remains an unanswered question.

Previous epidemiological studies have not answered this question since they have investigated the influence of occupational physical activities on health “in silos”, ie, the association of each occupational physical activity with back pain was examined in isolation from the other activities (15, 16). For example, time spent in sitting time and physical activities of various intensities during work are usually treated as separate variables in epidemiological investigations. Specifically within occupational research it is often assumed that one occupational physical activity, eg, sitting time, influences health outcomes independent of other occupational physical activities. However, occupational time spent in different physical activities are mutually exclusive components of a composition and constitute a finite total, ie, they sum up to 100% or 8 hours. That is, increasing time to one occupational physical activity will lead to the decrease of at least one remaining occupational physical activity. Consequently, it is the co-dependency of occupational physical activities that influence health outcome differently (17). Thus, to answer the above posed question, the present study follows an approach that investigates how different combinations of all occupational physical activities throughout a workday are associated with back pain. This knowledge can ultimately aid in formulating guidelines for eldercare workers that promote a healthy work environment.

A novel concept, termed the “sweet-spot” of physical activity has been developed (18). This concept goes beyond investigating the composition of occupational physical activities with highest risk for poor health (termed the “sour-spot”), to also investigating the composition of physical activities associated with highest probability for better health (18). In line with this concept, the German Committee on Occupational Safety and Health has designed a general guideline providing information about compositions of occupational physical activities associated with highest risk (“sour-spot”) as well as the “sweet-spot” associated with better health. The “sweet-spot” was defined as worktime spent with 60% sitting, 30% standing, and 10% walking/active (19). Thus, in line with the “sweet-spot” concept, the German Committee provides a guideline on how workers should aim to distribute their worktime between physical activities to decrease risk and promote health.

However, based on available literature, the German guideline is not based on empirical analyses of data using a prospective research design. Thus, there is no underlying empirical research supporting the notion that adhering to the German Committee guideline promotes health. Therefore, an empirical investigation is needed to investigate the validity and precision of the guideline. Furthermore, the guideline was formulated as a general recommendation for various manual occupations rather than being tailored to specific working populations. As different manual occupations might have different levels of physical activity due to specific job tasks related to the job, their “sweet-” and “sour-spot” might differ.

Thus, our aim was to investigate if there exists an occupational physical activity “sweet-spot” (lowest risk) and “sour-spot” (highest risk) for back pain among eldercare workers. Given that eldercare workers have a high prevalence of back pain along with the large inter-individual differences in occupational physical activity and constitute a rather homogenous group, we consider it an optimal occupational group to initiate the investigation of the “sweet-” and “sour-spot”. We hypothesized that eldercare workers whose worktime is characterized by 60% sitting, 30% standing, and 10% walking/active (“sweet-spot”) will have a lower risk of back pain compared to workers who deviate from this composition of occupational physical activities, particularly those having little worktime sitting, much worktime standing and much worktime with moderate-to-vigorous physical activity (“sour-spot”).

## Methods

### Study design and population

One-year longitudinal follow-up data were used from the Danish Observational Study of Eldercare work and musculoskeletal disorderS (DOSES) cohort. A comprehensive description of the recruitment strategy, study design, data collection, and methodology of DOSES has previously been published (20). The Danish Data Protection Agency and the Ethics Committee for the Capital Region of Denmark granted DOSES with ethical approval (H-4-2013-028) and written informed consent was obtained from all eldercare workers before their involvement in the study.

Eighty-three nursing homes located in the Zeeland region in the larger Copenhagen area in the eastern part of Denmark were invited to participate in the study. The nursing homes were selected in order to represent a mix of sizes and care models, ensuring inclusion of both smaller and larger facilities. In total 20 nursing homes, including 126 wards and 553 eldercare workers, agreed to participate and were included in the study. Two nursing homes were private, and 18 were municipal. Among the 553 eldercare workers agreed to participate 95% (N=525) were females as this professional group is predominantly female-dominated.

To be eligible to participate in DOSES, individuals had to meet the following criteria: be aged 18–65 years; work for ≥15 hours per week; have day, evening, or rotational shifts; and allocate ≥25% of their working time to activities directly related to resident care. Those who were on long-term sickness absence, pregnant, not in permanent employment, or did not spend ≥25% of their working time on tasks directly related to elderly care were excluded from the study. In this study, data from self-administered questionnaires, baseline 24-hour accelerometer-based measurements of physical activity, one-year follow-up SMS-based musculoskeletal pain outcomes and health checks were used.

### Accelerometry

Information about 24-hour accelerometer-based measurements of physical activity were collected from a total of 452 eldercare workers with a thigh-worn ActiGraph GT3X+ accelerometer (ActiGraph, Florida, USA). Eldercare workers were asked to wear the thigh-worn accelerometer for a minimum of four consecutive days including at least two working days. Work, leisure and time in bed were identified from the diaries that the eldercare workers were asked to complete during each day. The accelerometer data were processed with the MATLAB program Acti4, which has previously shown to have a high sensitivity and specificity (21). Specifically, Acti4 was used to identify time spent in various physical activities, including sitting (sitting and/or lying), standing still, moving (standing with slight movements), slow walking (<100 steps per minute), fast walking (≥100 steps per minute), running, cycling, and stair climbing. These physical activities were then combined into four categories: (i) sitting (sitting and / or lying), (ii) standing (standing still and / or standing with slight movements), (iii) light physical activity (slow walking) and (iv) moderate-to-vigorous physical activity (fast walking, climbing stairs, and/or running). Leisure-time moderate-to-vigorous physical activity also included cycling time.

For this analysis, all non-workdays as well as non-wear period were excluded. Eldercare workers who had ≥1 day with valid work or leisure-time were involved in the analysis. Valid periods of work or leisure-time were defined as those with wear time of ≥4 hours or comprising ≥75% of the average wear-time across days. A daily period of time spent in bed was defined as all sleep periods occurring within a 24-hour workday and used as a proxy for sleep time. Time in bed data were further checked for accuracy by visual inspection of the accelerometer data using Acti4. Time spent sitting, standing, on light physical activity, on moderate-to-vigorous physical activity and time in bed across all valid days during work and leisure-time for each individual were subsequently averaged to express 24-hour physical activity.

### Back pain intensity

Data on back pain intensity were collected at baseline and then every four weeks until one year from baseline (22). Via SMS, eldercare workers were asked to report their low-back pain intensity (measured on an 11-point numeric rating scale with 0 indicating no pain and 10 indicating the worst pain imaginable). The one-year follow-up data on low-back pain intensity were used as the outcome, while the baseline pain intensity data were used as a confounder as done previously (23).

### Covariates

We chose potential confounders a priori. Data on selected confounders were collected at baseline via a questionnaire or a health check. The sex of the workers was determined by using a single item (“Are you male of female?”). Body mass index (BMI) was determined objectively by measuring their height (cm) and weight (kg). Smoking status was determined using a single item with response categories summarized to smokers (smoking daily or sometimes) and non-smokers (ex-smokers and never smoked). Baseline values of back pain intensity were collected with a single item questions (“What was your worst pain in the low back within the last four weeks?”) with possible responses ranging 0–10. Occupational position was assessed through payroll with the following categories: (i) helper; (ii) assistant, (iii) carer / care assistant, (vi) home care assistant, nursing care assistant, nursing home assistant, (v) uneducated caregiver, (vi) other, (vii) nurses, (viii) social pedagogue, pedagogue and care worker, (ix) leader, and (x) social/psychiatric dementia team employee. Categories iii–x where merged together to constitute “others”. Seniority was determined with a single item question (“How many months have you been working in your current position?”). Lastly, we determined the average number of repositions, turnings and transfers required for each nursing home resident by conducting 4716 observations during morning and evening shifts aggregated at the ward level and linked them with all eldercare workers according to the ward they were working at.

### Statistical analysis

*Main analysis - identifying the “sweet- and sour-spot” of occupational physical activity for back pain.* 24-hour accelerometer-based measurements of daily occupational (sitting, standing, light physical activity and moderate-to-vigorous physical activity) and leisure-time (sitting, standing, light physical activity, moderate-to-vigorous physical activity and time in bed) physical activities are compositional by nature. The composition of various daily physical activities at work and leisure for each participant sum up to 100% of its time. Additionally, compositional data are inherently codependent (ie, increasing time spent within the daily occupational composition in one of the physical activities will lead to the compensatory decrease of one or more remaining activities in order to maintain a total of 8 hours). To handle such data, we used the recommended compositional data analysis (CoDA) approach (24). For an easy understanding of how CoDA is utilized in occupational research, please refer to the explanation provided here (25). Occupational and leisure-time physical activity compositions were treated as two separate sub-compositions of a 24-hour day. Occupational and leisure-time physical activity compositions were expressed as a set of isometric log-ratio (ilr) coordinates (see supplementary material www.sjweh.fi/article/4170, appendix 1 for the calculation of ilrs).

Back pain data were checked for normality and were found to depict a high proportion of zeros (ie, zero-inflation) and a right-skewed distribution (supplementary figure S1). This was further checked by initially fitting a Poisson model and checking for zero-inflation. The test indicated that the Poisson model was underfitting zeros as the amount of observed zeros is larger than the amount of predicted zeros for the back pain intensity data (ie, presence of zero-inflation). Previous research on statistical models for analyzing pain intensity data has revealed that zero-inflated generalized mixed-effects models either with a generalized Poisson or a negative binomial distribution (originally designed for count data), outperformed other statistical models in terms of providing a superior fit to the data (26). Thus, zero-inflated generalized mixed-effects models with a generalized Poisson distribution and a negative binomial distribution (with a linear and a quadratic variance increase) were fit to examine the longitudinal association between occupational physical activity and back pain intensity. Due to convergence issues in the models utilizing the negative binomial distribution, the final model was the one with the generalized Poisson distribution. Post-model-fitting diagnostic plots indicated a non-linear relationship between the occupational physical activity (expressed as ilrs) and the back pain intensity. Subsequently, a quadratic term for the occupational physical activity was tested and retained if it improved the model fit (partial F test for nested models P<0.05). The model was adjusted for leisure-time physical activity (expressed as ilrs), sex, BMI, smoking, baseline values of back pain intensity, occupational position and seniority. Leisure-time physical activity was included in the model as it has been shown to be associated with back pain and being inter-dependent of occupational physical activity (14, 27). To account for the hierarchical structure of the data (workers within wards) the model used a mixed-effects structure that included workers and wards as random intercepts in the analysis. The significance of the occupational physical activity composition was assessed using Wald chi-squared ANOVA tables with type II test for both the conditional and the zero-inflated part of the generalized mixed-effects model. The significance level was set at P<0.05.

Thereafter, a predictive grid with hypothetical occupational physical activity compositions (in 5-minute increments) was created within the measured limits of all occupational physical activities. The limits of each physical activity were truncated at ±3 standard deviations (SD) of their univariate distribution to avoid values that could be potentially implausible. Each hypothetical composition in the grid that summed to the sample’s observed average total measured occupational time of 428.2 minutes was selected for further analysis. This resulted in a total of 6506 unique hypothetical compositions that were then transformed to ilrs (as explained above). The ranges for all chosen hypothetical compositions were: sitting = 10–385, standing = 10–315, light physical activity = 5–45, and moderate-to-vigorous physical activity = 5–110 minutes/day.

The model estimates from the aforementioned fitted zero-inflated generalized mixed-effects model were then used on the grid to predict back pain intensity associated with each hypothetical composition. In this prediction, we used the average values of the continuous confounders (leisure-time physical activity composition expressed as a set of ilrs, BMI, seniority and baseline values of back pain). For the categorical confounders, we used the most prevalent category observed in our study population (ie, sex = female, smoking = non-smoker, position = health service helper). For these predictions, all random effects (worker and ward level) were set to zero as the aim was to compute population-based predictions.

The occupational physical activity compositions associated with lowest and highest 5% of the predicted values of back pain were considered to be the “sweet- and sour-spot”, respectively. The “sweet- and sour-spot” compositions were described by means (minutes/day), range (minutes/day) and percentage of occupational time. The occupational physical activity compositions associated with each residual 5% predicted back pain values in between the “sweet- and sour-spot” were defined as 5% occupational time zones of physical activity. Further, the mean of each occupational time zone of physical activity compositions associated with each 5% of predicted back pain was plotted in an interactive quaternary tetrahedron plot (4-simplex).

Previous research has suggested a change of 2 units in back pain intensity on the scale of 0–10 to be of clinical relevance (28). Thus, apart from statistical significance, the “sweet- and sour-spot” were considered clinically relevant if the difference in the predicted back pain intensity between them was ≥2 units on the scale of 0–0.

All statistical analysis was conducted in R software using the ‘compositions’ (29) and ‘glmmTMB’ packages (30). The visualization of occupational physical activity compositional means with the quaternary tetrahedron plot (4-simplex) was performed with the ‘complexity’ package.

### Sensitivity analysis

To test the sensitivity of the results obtained from the main analysis, we performed two additional analyses. The observed association in the main analysis could be confounded by the number of repositions, turnings and transfers of nursing home residents by the eldercare workers. Thus, we performed a sensitivity analysis where we also adjusted for the average number of repositions, turnings and transfers required for each nursing home residents during day and evening shifts aggregated at the ward level. We also performed a separate analysis where we excluded BMI from the covariates in order to test if BMI could act as a potential confounder.

## Results

### Main analysis

Of the 553 eldercare workers who agreed to participate, 452 provided 24-hour accelerometer data, of which 396 were included in this study as they had valid data of at least one working day and replied to the SMS query about back pain intensity (supplementary figure S2). These 396 eldercare workers were on average 45.9 (SD 10.5) years old, had an average BMI of 26.3 (SD 5.2) kg/m^2^ and had a seniority of 194.9 (SD 131.7) months. Additionally, 95.7% were women, 35.4% were smokers and 45.7% were employed as health service helpers. The average low-back pain (on 0–10 scale) was 3.9 (SD 3.3) and 3.4 (SD 3.1) for the baseline and follow-up period, respectively. The characteristics of the sample can be seen in table 1.

**Table 1:** General characteristics of the eldercare workers participating in the study (N=396). [SD=standard deviation.]

Characteristics		N (%)	Mean (SD)
Age (years)			45.9 (10.5)
Female		379 (95.7)	
Danish		311 (78.5)	
BMI			26.3 (5.2)
Smokers		140 (35.4)	
Position			
	Helper	181 (45.7)	
	Assistant	162 (40.9)	
	Other	53 (13.4)	
Type of ward			
	Somatic	87 (74.4)	
	Dementia	25 (21.4)	
	Rehabilitation	3 (2.6)	
	Psychiatric	2 (1.7)	
Seniority (months)			194.9 (131.7)
Shift			
	Day	231 (58.3)	
	Evening	85 (21.5)	
	Day / evening	18 (4.5)	
	Day / evening / night	62 (15.7)	
Working hours (per week)			32.4 (3.6)
Staff ratio during day shifts (worker per residents)			0.31 (0.07)
Staff ratio during evening shifts (worker per residents)			0.15 (0.06)
Repositions, turnings and transfers of residents (per ward)			0.5 (0.3)
Back pain intensity - baseline			3.9 (3.3)
Back pain intensity - follow-up			3.4 (3.1)

On average, the eldercare workers wore the accelerometers for 7.1 (SD 1.1) hours at work, 8.7 (SD 1.2) hours during leisure and 7.0 (SD 1.1) hours during time in bed, summing to a total of 22.8 (1.6 SD) hours per day. Furthermore, the eldercare workers were on average measured for 3.2 (1.3) workdays, for 3.2 (SD 1.3) leisure days and for a total of 3.7 (SD 1.3) days (table 2).

**Table 2:** Accelerometer-based measurements of physical activities at work and leisure over the workdays for the participating eldercare workers (N=396). [SD=standard deviation.]

Time spent on physical activity (minutes)		% ^a^	Mean (SD)
Work			
	Sitting (minutes/day)	38	161.9 (56.9)
	Standing (minutes/day)	46	195.2 (49.7)
	Light physical activity (minutes/day)	2	11.2 (6.8)
	Moderate-to-vigorous physical activity (minutes/day)	14	59.9 (18.6)
Leisure			
	Sitting (minutes/day)	34	321.5 (78.2)
	Standing (minutes/day)	15	142.1 (47.8)
	Light physical activity (minutes/day)	1	9.9 (6.1)
	Moderate-to-vigorous physical activity (minutes/day)	5	47.3 (22.4)
	Time in bed (minutes/day)	46	420.6 (63.2)
Mean measured time per day work (minutes/day)			428.2 (66.4)
Mean measured time per day leisure (minutes/day)			941.5 (80.2)
Total measured days			3.7 (1.3)
Total measured work days			3.2 (1.3)
Total measured leisure days			3.2 (1.3)

^a^ Represents that these activities of the occupational and leisure composition are presented as a proportion of the 24-hour day.

**Figure 1:**
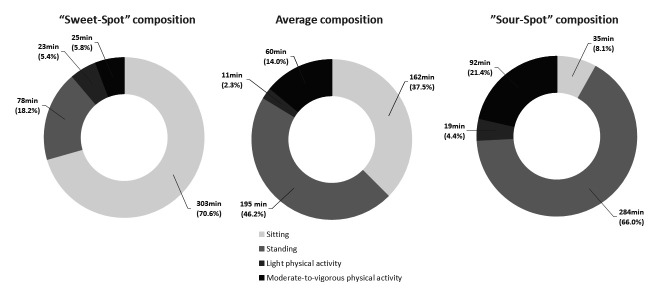
Pie chart of the compositional mean and percentage of occupational physical activity of the “average” of the participants”, the “sweet-spot” (associated with the lowest 5% of predicted back pain) and “sour-spot” (associated with the highest 5% of predicted back pain) of eldercare workers (N=396).

Figure 1 visualizes (i) the “sweet-spot” associated with 5% lowest risk for back pain, (ii) the average composition of the study population and (iii) the “sour-spot” associated with 5% highest risk for back pain.

The average composition of occupational physical activity among this group of eldercare workers consisted of 162 minutes (38%) worktime spent sitting, 195 minutes (46%) spent standing, 11 minutes (2%) spent on light physical activity, and 60 minutes (14%) spent on moderate-to-vigorous physical activity.

The “sweet-spot” of occupational physical activity composition associated with the lowest 5% back pain consisted of more sitting and light physical activity time and less standing and moderate-to-vigorous physical activity time compared to the average composition. Specifically, the compositional mean, range (min–max) and percentage contribution of the “sweet-spot” consisted of 303 (235–330) minutes (71%) worktime spent sitting; 78 (50–160) minutes (18%) spent standing; 23 (5–30) minutes (5%) spent on light physical activity; and 25 (5–95) minutes (6%) spent on moderate-to-vigorous physical activity.

**Figure 2:**
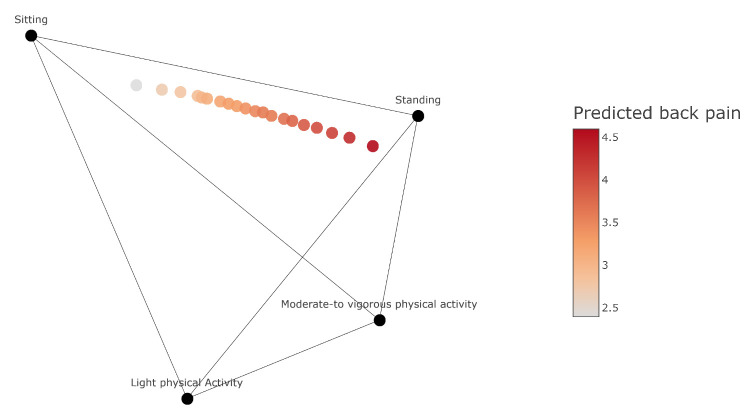
Quaternary tetrahedron plot (4-simplex) depicting the occupational physical activity compositions from the lowest 5% (“sweet-spot”; grey shade) to the highest 5% (“sour-spot”; red shade) of predicted back pain among eldercare workers (N=396). Each data point sums up to the total measured worktime of occupational physical activity (428.2 minutes or 7.1 hours). Each component of physical activity (ie, sitting, standing, light physical activity and moderate-to-vigorous physical activity) is at its 100% (7.1 hours) duration at each corresponding vertex of the plot and gradually decreasing to 0% (0 hours) while moving towards the opposite direction. An example on how to read a compositional mean: Each data point indicates that moving towards the sitting and light physical activity vertexes and away from the standing and moderate-to-vigorous physical activity vertexes is expected to reduce the predicted back pain by ≈ 0.1. Back pain of the sample was measured on a scale from 0–10. However, the predicted back pain of each occupational physical activity composition ranges between 2.4–4.6 which is shown on the plot’s legend. For an interactive quaternary tetrahedron plot please visit: https://chart-studio.plotly.com/~stavros_kyriakidis/3/#/. For the percentage of sitting, standing, light physical activity and moderate-to-vigorous physical activity and the predicted back pain intensity of each occupational physical activity composition please refer to the interactive plot. Results are adjusted for leisure-time physical activity composition, sex, body mass index, smoking, baseline values of back pain, occupational position and seniority.

The “sour-spot” of occupational physical activity composition associated with the highest 5% back pain consisted of more standing and moderate-to-vigorous physical activity time and less sitting and light physical activity time compared to the average composition. The compositional mean, range (min–max) and percentage contribution of the “sour-spot” consisted of 35 (15–65) minutes (8%) worktime spent sitting; 284 (235–310) minutes (66%) spent standing; 19 (5–30) minutes (4%) spent on light physical activity; and 92 (60–105) minutes (21%) spent on moderate-to-vigorous physical activity (figures 1&2 and supplemenary table S1).

The results from the model indicated that the overall composition of occupational physical activity was not statistically significantly associated with back pain [conditional model (chi-squared test 4.95, P=0.18); zero-inflated model (chi-squared test 3.82, P=0.28). The regression coefficients obtained from the model for each individual ilr can be found in table 3. However, there was a clinically significant difference of 2.2 in the predicted back pain intensity (on 0–10 scale) between the “sweet-spot” of occupational physical activity composition (predicted back pain intensity = 2.4) and the sour-spot” of occupational physical activity composition (predicted back pain intensity = 4.6).

**Table 3:** Regression estimates obtained from the zero-inflated generalized linear mixed-effects models to investigate the association between occupational physical activities (sitting, standing, light physical activity and moderate-to-vigorous physical activity) and 1 year-follow up risk of back pain intensity. Interpretation of the coefficients: The conditional and zero-inflated model work on the opposite direction. The zero-inflated model estimates the probability of an extra zero such that a positive contrast (OR >1.00) indicates a higher chance of having zero back pain intensity. This is the opposite of the conditional model where a positive contrast (RR >1.00) indicates a higher risk of back pain intensity. [CI=confidence interval; IIr=isometric log-ratio; OR=odds ratio; RR=relative risk.]

Variables	Dependent variable: Back pain intensity						
	Conditional model				Zero-inflation model		
	RR	95% CI	P-value		OR	95% CI	P-value
Ilr1	0.95	0.98– 1.03	0.20		1.61	0.84– 3.10	0.15
Ilr2	0.84	0.71– 1.01	0.07		0.45	0.10– 2.05	0.30
Ilr3	0.95	0.85– 1.06	0.35		1.20	0.47– 3.03	0.70

### Sensitivity analysis

The results of the main [conditional model (chi-squared test 4.95, P=0.18); zero-inflated model (chi-squared test 3.82, P=0.28) and sensitivity analysis [conditional model (chi-squared test 5.04, P=0.17); zero-inflated model (chi-squared test 3.03, P=0.39) were similar when further adjusting the primary model for the average number of repositions, turnings and transfers required for the nursing home residents during day and evening shifts aggregated at the ward level (supplementary tables S2 & S3). When excluding BMI from the primary model results also remained similar suggesting a small confounding effect (supplementary tables S4 & S5). However we included BMI in the model as there are theoretical grounds of BMI being associated with both physical activity and back pain.

## Discussion

To the best of our knowledge, this is the first prospective study investigating the “sweet and sour-spot” of occupational physical activity related to back pain in an occupational population. By this means, we could make a first empirical exploration of the advice from the German Committee on Occupational Safety and Health of the “sweet-spot”. Our results of the “sweet-spot” for lowest risk of back pain are not directly in line with the guideline from the German Committee on Occupational Safety and Health in which workers are advised to spend 60% of their occupational time sitting, 30% standing, and 10% walking/active. We found the average composition of occupational physical activity among eldercare workers to be 38% sitting, 46% standing, 2% in light physical activity, and 14% in moderate-to-vigorous physical activity. The “sweet-spot” consisted of 71% of worktime sitting, 18% standing, 5% in light physical activity and 6% in moderate-to-vigorous physical activity (total 11% walking / active). The “sour-spot” consisted of 8% of worktime sitting, 66% standing, 4% in light physical activity and 21% in moderate-to-vigorous physical activity (total 25% walking / active).

In our study, we found that increasing the durations of occupational sitting and light physical activity while minimizing time spent on standing and moderate-to-vigorous physical activity tended to bring individuals closer to the “sweet-spot” for lowest risk of back pain. On the contrary, decreasing the durations of occupational sitting and light physical activity tended to bring the individuals closer to the “sour-spot”. In accordance, the “sour-spot” – was characterized by longer durations of standing and moderate-to-vigorous physical activity – and this deviates significantly from the advised 60% sitting, 30% standing, and 10% walking/active issued by the German Committee on Occupational Safety and Health. These discrepancies shed light on the potential discord between the guideline and our study’s findings. One possible explanation for the observed discrepancy between our findings and the German guideline could be that the guideline was not based on research of empirical data, but experts’ opinion and consensus. However, it’s not uncommon for guidelines on risk factors and prevention to be formulated based on experts’ opinion and consensus without strong empirical support from research. Furthermore, another reason for the observed difference could be that the guideline was formulated for the general occupational population, while our findings are limited to eldercare workers. Thus, we cannot conclude that the “sweet-” and “sour-spot” differs between occupations. Therefore, it remains unclear if the German guideline fits well with the general occupational population. However, it is essential to acknowledge that our study represents an initial exploration, focusing on a single occupation and a single outcome. Thus, more studies need to be conducted exploring the German Committee’s guideline before any modifications can be considered.

The results of this study have implications in the development of evidence-based guidelines for organizing eldercare work towards the “sweet-spot” of occupational physical activity for lowest risk of back pain. Furthermore, the identified “sour-spot” can be useful in complementing the guidelines in terms of which detrimental occupational physical activities to “move away from”. By utilizing our results of the “sweet- and sour-spot” of occupational physical activity of eldercare workers, practitioners and policymakers can create healthier and more productive work environments. However, it is also important to consider the feasibility of implementing the suggested “sweet-spot” of occupational physical activity in the eldercare work environment. Given that many of the tasks involved in eldercare work require standing and walking, it is necessary to evaluate whether the recommended “sweet-spot” of occupational physical activity is compatible with the demands of the job and does not interfere with the completion of essential tasks. This could be accomplished through carefully designed feasibility studies that assess the impact of the recommended “sweet-spot” of occupational physical activity on work performance.

The results related to the “sweet- and sour-spot” were not statistically significant. Reasons for non-statistical significant results pertain to possible individual differences around the “sweet-” and “sour-spot” which could hinder the detection of an association (31). To address this challenge, future studies should include a large sample size that covers a wide range of the exposure and outcome that could possibly yield more robust and significant results. Nevertheless, that does not preclude that the observed “sweet-spot” for reduced back pain was not of relevance as the insignificant P-value does not provide any statement about the predictive ability of the model rather than a measure of strength against the hypothesis that a given coefficient is zero. On the contrary, the root mean square error, a metric for the predictive accuracy of our model, was low (ie, 1.9) indicating good predictive ability. Since this study was explorative in nature and conducted on a small dataset, similar future cohorts are needed where such “sweet-spot” of occupational physical activity can be intervened with enough statistical power and longer follow-up period.

Besides statistical significance, we further assessed if our results were clinically relevant. We observed a 2.2 difference in back pain intensity score (0–10 scale) between the 5% lowest (“sweet-spot”) and highest (“sour-spot”) predicted back pain of occupational time zones of physical activities. It has been suggested that the results are of clinical significance if reduction in the pain intensity score is ≥2 points on a scale of 0–10 (28). Thus, we believe that the observed “sweet-spot” should be considered clinically significant for improvement of back pain among eldercare workers (supplementary table S1). To achieve a 2.2-unit change from the “sour-spot” to the “sweet-spot”, it is essential to consider feasible workplace adjustments for eldercare workers. The key to the success of this new approach lies in bringing eldercare workers closer to the “sweet-spot” rather than rigidly adhering to those specific cut-off points of occupational physical activity during a working day. A practical strategy for achieving this involves, for example, adapting certain activities that typically involve standing into activities that can be done seated (eg, preparing medication for residents, feeding them or assisting in putting on their socks).

### Strengths and limitations

One of the major strengths of this study is the 24-hour accelerometer-based measurements of physical activities. Furthermore, the use of novel analytical methods (CoDA), which allowed to simultaneously examine the collective effect of all occupational physical activities is another strength. Longitudinal 1-year repeated collection of back pain data minimized any potential issue of reverse causality and recall bias. Zero-inflated generalized mixed-effects models were used to model pain intensity data, which often present a large proportion of zeros making statistical models that assume linearity inappropriate. Finally, the multilevel design offered the unique possibility to consider the effect of hierarchical levels in the DOSES cohort.

In spite of these strong features, the study also suffers from some limitations. These include that the categorization of individual physical activities relies on the analysis of postures and movements captured by accelerometers, rather than directly measuring the intensity of each individual activity and classifying it accordingly. Furthermore, another limitation is that we used data on the observed number of repositions, turnings and transfers required for each nursing home resident aggregated at the ward level and not on the observed number that were actually performed by each eldercare worker. While this method served as a proxy for the number of repositions, turnings and transfers each worker performed, it has limitations and may be imprecise due to it not being directly derived from the eldercare workers. Future studies should aim to collect individual-level data to more accurately estimate the number of repositions, turnings and transfers. In addition, our results might be limited by task-specificity related to eldercare work. However, a stratified analysis by occupational position (ie, helper, assistant and others) was not possible due to the small sample size of the “others” category (N=53) which would reduce statistical power and limit the precision of our results. A diary-based detection of time in bed was used as a proxy for sleep. Such self-reported measures do not distinguish between wakeful and non-wakeful time. Recent advancements of sleep estimation algorithms using thigh-based accelerometer has lately been developed with sufficient accuracy (32). Thus, future research should implement this as a valid tool for measuring sleep. Lastly, our sample of Danish population from the administrative region of Zealand and the Capital Region of Denmark might limit the generalizability of our results. However, predictions were made for the general population of eldercare workers conditioning on the random effects (ie, the hypothetical occupational physical activity compositions do not come from the observed group of eldercare workers) which makes the results more generalizable.

### Concluding remarks

This study is the first attempt to uncover the “sweet- and sour-spot” of occupational physical activity for back pain in eldercare workers. The “sweet-spot” of occupational physical activity for back pain among eldercare workers involves more sitting and light physical activity time and less standing and moderate-to-vigorous physical activity time than recently recommended by the German Committee on Occupational Safety and Health. On the contrary, the “sour-spot” is characterized by more standing and moderate-to-vigorous physical activity time and less sitting and light physical activity time. These findings offer valuable insights for better promotion and prevention strategies for eldercare workers in terms of providing knowledge towards the physical activity composition to strive for (“sweet-spot”) and to avoid (“sour-spot”), respectively. However, more practical tests are needed on the possibilities of implementing a move from “sour-spot” to “sweet-spot” and if it actually offers an improvement in back pain to a higher degree than the pre-existing guideline.

## Supplementary material

Supplementary material
