# Supplementary material for: The “sweet- and sour-spot” of occupational physical activity for back pain: a prospective accelerometer study among eldercare workers
Source: Scand J Work Environ Health. 2024 Jun 27;50(5):341–50. doi: 10.5271/sjweh.4170 (PMC11239222; doi:10.5271/sjweh.4170)
Supplement: Supplementary material [file SJWEH-50-341-S001.pdf]

The "sweet- and sour-spot" of occupational physical activity for back pain: a prospective accelerometer study among eldercare workers[1]

by Stavros Kyriakidis, MSc,[2] Charlotte Lund Rasmussen, PhD, Karen Søgaard, PhD, Andreas Holtermann, PhD, Charlotte Diana Nørregaard Rasmussen, PhD, Nidhi Gupta, PhD

1. *Supplementary material*
2. *Correspondence to: Stavros Kyriakidis, National Research Centre for the Working Environment, Lersø Parkallé 105, 2100 København Ø, Copenhagen, Denmark. [E-mail: stk@nfa.dk]*

Appendix 1. Calculations of ilr coordinates for the occupational and leisure-time physical activities

$$ilr1_{work_i} = \sqrt{\frac{3}{4}}\ln\left(\frac{Sedentary_{work_i}}{\sqrt[3]{Standing_{work_i} * LPA_{work_i} * MVPA_{work_i}}}\right)$$

$$ilr2_{work_i} = \sqrt{\frac{2}{3}}\ln\left(\frac{Standing_{work_i}}{\sqrt[2]{LPA_{work_i} * \; MVPA_{work_i}}}\right)$$

$$ilr3_{work_i} = \sqrt{\frac{1}{2}}\ln\left(\frac{LPA_{work_i}}{MVPA_{work_i}}\right)$$

$$ilr1_{leis_i} = \sqrt{\frac{4}{5}}\ln\left(\frac{Sedentary_{leis_i}}{\sqrt[4]{Standing_{leis_i} * LPA_{leis_i} * MVPA_{leis_i} * Time\ in\ bed_{leis_i}}}\right)$$

$$ilr2_{leis_i} = \sqrt{\frac{3}{4}}\ln\left(\frac{Standing_{leis_i}}{\sqrt[3]{LPA_{leis_i} * MVPA_{leis_i} * Time\ in\ bed_{leis_i}}}\right)$$

$$ilr3_{leis_i} = \sqrt{\frac{2}{3}}\ln\left(\frac{LPA_{leis_i}}{\sqrt[2]{MVPA_{leis_i} * Time\ in\ bed_{leis_i}}}\right)$$

$$ilr4_{leis_i} = \sqrt{\frac{1}{2}}\ln\left(\frac{MVPA_{leis_i}}{Time\ in\ bed_{leis_i}}\right)$$

Where i is one worker

The composition of occupational and leisure-time physical activities was transformed to isometric log ratios (ilr), which allows the compositional parts to be used in standard statistical procedures. More specifically, the first ilr coordinate for the occupational and leisure-time physical activities compositions represents time spent sedentary relative to the geometric mean of remaining activities. For the residual ilrs, the denominator of the first ilr was further split to create the rest of the ilrs.

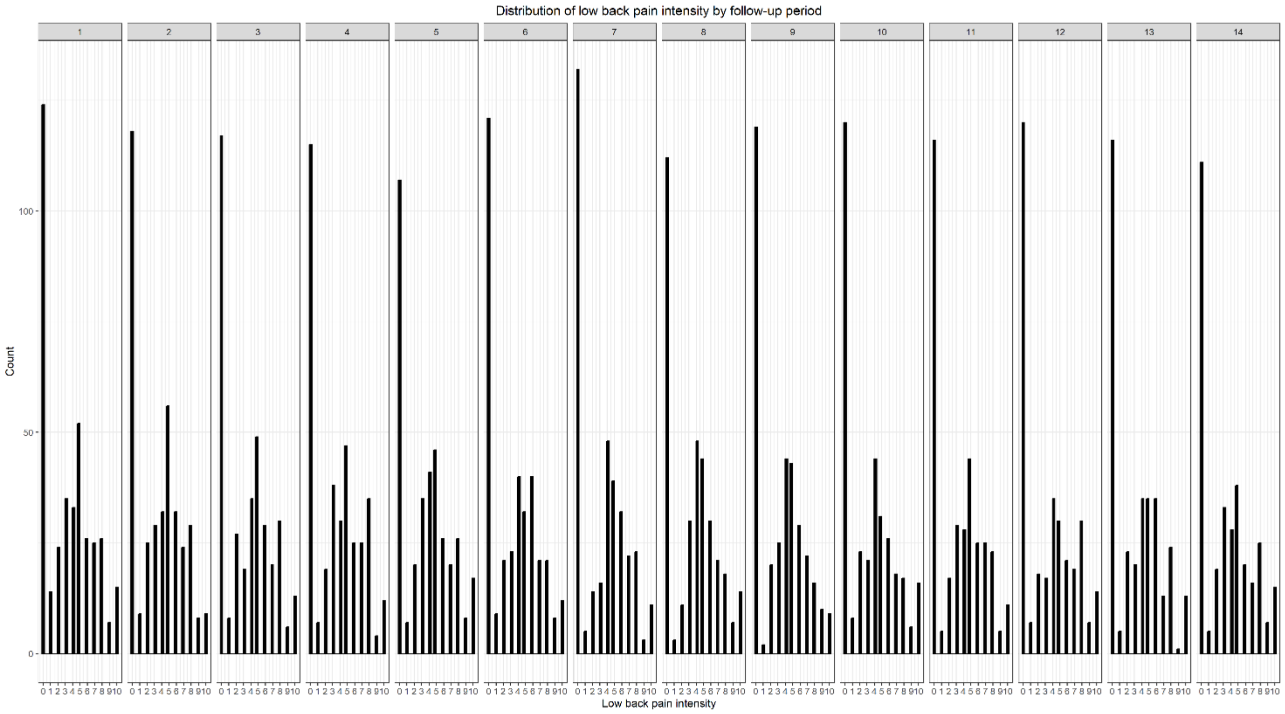

Figure S1. Distribution of LBP intensity by follow-up period

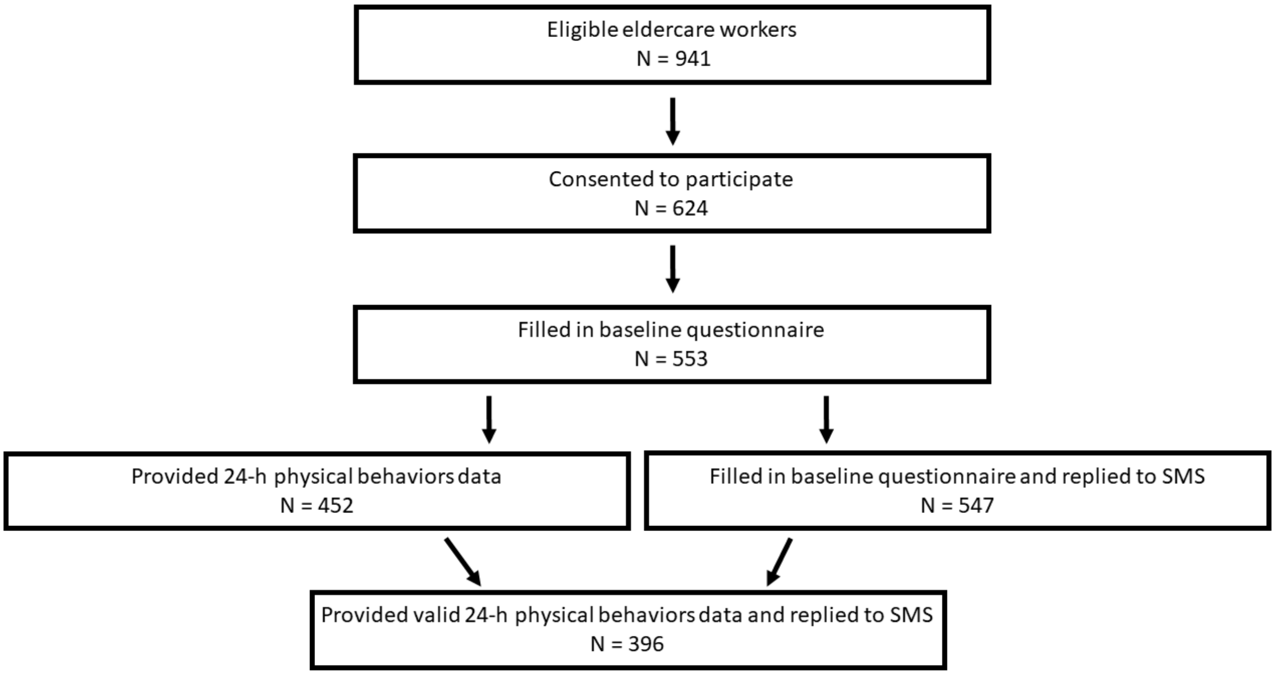

Figure S2. Flow chart of the participants in the study

Table S1. Zones of occupational physical activities compositions ranked from the “Sweet-” to “Sour-Spot”

Zones of occupational physical activities	Sedentary mean [range] (min / d)[a] (% of work-time)	Stand mean [range] (min / d)[a] (% of work-time)	Light physical activity mean [range] (min / d)[a] (% of work-time)	Moderate-to-vigorous mean [range] (min / d)[a] (% of work-time)	Predicted LPB intensity (0 - 10)	95% CI
0 - 5%	303 [235; 330] (70.6%)	78 [50; 160] (18.2%)	23 [5; 30] (5.4%)	25 [5; 95] (5.8%)	2.4	0 - 8.2
5 - 10%	277 [190; 330] (64.3%)	100 [50; 205] (23.2%)	20 [5; 30] (4.6%)	34 [5; 105] (7.9%)	2.7	0 - 8.3
10 - 15%	258 [150; 330] (60.1%)	118 [50; 250] (27.5%)	18 [5; 30] (4.2%)	35 [5; 105] (8.2%)	2.8	0 - 8.2
15 - 20%	241 [110; 320] (55.9%)	135 [50; 310] (31.3%)	18 [5; 30] (4.2%)	37 [5; 105] (8.6%)	2.9	0 - 8.1
20 - 25%	235 [90; 310] (54.7%)	137 [50; 310] (31.9%)	18 [5; 30] (4.2%)	40 [5; 105] (9.3%)	3.0	0 - 8.2
25 - 30%	229 [85; 300] (53.3%)	141 [50; 310] (32.8%)	17 [5; 30] (4%)	43 [5; 105] (10%)	3.1	0 - 8.2
30 - 35%	215 [105; 290] (50%)	154 [55; 310] (35.8%)	17 [5; 30] (4%)	44 [10; 105] (10.2%)	3.2	0 - 8.1
35 - 40%	205 [85; 280] (47.8%)	161 [60; 310] (37.5%)	17 [5; 30] (4%)	46 [10; 105] (10.7%)	3.3	0 - 8.1
40 - 45%	196 [80; 270] (45.6%)	168 [65; 310] (39.1%)	17 [5; 30] (4%)	49 [10; 105] (11.4%)	3.3	0 - 8.1
45 - 50%	186 [85; 260] (43.3%)	176 [70; 310] (40.9%)	17 [5; 30] (4%)	51 [15; 105] (11.9%)	3.4	0 - 8.1
50 - 55%	175 [75; 245] (40.7%)	184 [80; 310] (42.8%)	17 [5; 30] (4%)	54 [15; 105] (12.6%)	3.5	0 - 8.1
55 - 60%	166 [75; 235] (38.7%)	190 [85; 310] (44.3%)	16 [5; 30] (3.7%)	57 [20; 105] (13.3%)	3.6	0 - 8.1
60 - 65%	156 [70; 225] (36.2%)	197 [95; 310] (45.7%)	17 [5; 30] (3.9%)	61 [20; 105] (14.2%)	3.6	0 - 8.1
65 - 70%	142 [65; 210] (32.9%)	209 [110; 310] (48.5%)	17 [5; 30] (3.9%)	63 [25; 105] (14.6%)	3.7	0 - 8.0
70 - 75%	132 [60; 200] (30.7%)	215 [120; 310] (50%)	16 [5; 30] (3.7%)	67 [30; 105] (15.6%)	3.8	0 - 8.0
75 - 80%	118 [60; 180] (27.4%)	225 [140; 310] (52.2%)	17 [5; 30] (3.9%)	71 [35; 105] (16.5%)	3.9	0 - 8.0
80 - 85%	103 [50; 160] (24%)	236 [160; 310] (54.9%)	16 [5; 30] (3.7%)	75 [35; 105] (17.4%)	4.0	0 - 8.0
85 - 90%	85 [45; 135] (19.7%)	249 [185; 310] (57.8%)	17 [5; 30] (3.9%)	80 [45; 105] (18.6%)	4.1	0.3 - 8.0
90 - 95%	64 [35; 100] (14.9%)	265 [210; 310] (61.6%)	17 [5; 30] (4%)	84 [50; 105] (19.5%)	4.3	0.6 - 8.0
95 - 100%	35 [15; 65] (8.1%)	284 [235; 310] (66%)	19 [5; 30] (4.4%)	92 [60; 105] (21.4%)	4.6	1.1 - 8.2

LBP = Low back pain

Total % of zones of occupational physical activities compositions does not always sum up to 100% due to rounding

Table S2. Results of the regression estimates of the sensitivity analysis adjusted for repositions, turnings and transfers of nursing home residents during day and evening shifts aggregated at the ward level

	Dependent variable					
	**Back pain intensity**					
	Conditional model			Zero-inflation model		
**Variables**	Relative Risk	95% CI	P value	Odds Ratio	95% CI	P value
Ilr1	0.95	0.89 – 1.03	0.23	1.55	0.81 – 2.97	0.19
Ilr2	0.84	0.70 – 1.00	0.06	0.50	0.11 – 2.23	0.36
Ilr3	0.95	0.85 – 1.06	0.41	1.14	0.45 – 2.89	0.78

Table S3. Results of the ANOVA estimates of the sensitivity analysis adjusted for repositions, turnings and transfers of nursing home residents during day and evening shifts aggregated at the ward level

	Dependent variable			
	**Back pain intensity**			
	Conditional model		Zero-inflation model	
	Chisq	Pr(>Chisq)	Chisq	Pr(>Chisq)
Occupational composition	5.04	0.17	3.03	0.39

Table S4. Results of the regression estimates of the sensitivity analysis excluding BMI from the primary model

	Dependent variable					
	**Back pain intensity**					
	Conditional model			Zero-inflation model		
**Variables**	Relative Risk	95% CI	P value	Odds Ratio	95% CI	P value
Ilr1	0.96	0.90 – 1.04	0.32	1.57	0.84 – 2.97	0.16
Ilr2	0.83	0.69 – 0.98	**0.03**	0.54	0.12 – 2.39	0.42
Ilr3	0.94	0.84 – 1.05	0.32	1.28	0.51 – 3.22	0.60

Table S5. Results of the ANOVA estimates of the sensitivity analysis excluding BMI from the primary model

	Dependent variable			
	**Back pain intensity**			
	Conditional model		Zero-inflation model	
	Chisq	Pr(>Chisq)	Chisq	Pr(>Chisq)
Occupational composition	5.51	0.14	3.50	0.32
